# Early Preeclampsia Effect on Preterm Newborns Outcome

**DOI:** 10.3390/jcm11020452

**Published:** 2022-01-17

**Authors:** Melinda Matyas, Monica Hasmasanu, Ciprian N. Silaghi, Gabriel Samasca, Iulia Lupan, Kovacs Orsolya, Gabriela Zaharie

**Affiliations:** 1 Department of Neonatology, Iuliu Hatieganu University of Medicine and Pharmacy, 400006 Cluj-Napoca, Romania; melinda.matyas@umfcluj.ro (M.M.); Popa.Monica@umfcluj.ro (M.H.); gzaharie@umfcluj.ro (G.Z.); 2 Department of Biochemistry, Iuliu Hatieganu University of Medicine and Pharmacy, 400006 Cluj-Napoca, Romania; silaghi.ciprian@umfcluj.ro; 3 Department of Immunology, Iuliu Hatieganu University of Medicine and Pharmacy, 400006 Cluj-Napoca, Romania; 4 Interdisciplinary Institute in Bio-Nano-Science, 400006 Cluj-Napoca, Romania; iulia.lupan@ubbcluj.ro; 5 Department of Neonatology, County Clinical Emergency Hospital, 400006 Cluj-Napoca, Romania; kovacs_solya@yahoo.com

**Keywords:** preeclampsia, newborn, prematurity, respiratory distress, intraventricular hemorrhage

## Abstract

Background: An early form of preeclampsia is rare. Abnormal placentation, placental perfusion disorders, and inflammatory cytokine release will have an effect on the fetus and newborn. Material and methods: The study group consisted of preterm newborns whose mothers had a history of preeclampsia and a gestational age of between 30 weeks and 34 weeks + 6 days. The control group consists of neonates matched for gestational age with the case group, whose mothers had normal blood pressure. The incidence and severity of respiratory distress syndrome (RDS), intraventricular hemorrhage, hypoglycemia, pH gas changes, and hematological parameters were analyzed in the two groups. Results: The study group of preterm neonates had a lower birth weight than the control group (*p* < 0.001). Most of the deliveries in the group of newborns exposed to preeclampsia were performed by cesarean section. Severe forms of RDS were two times more frequent in the group of newborns exposed to preeclampsia compared to those in the control group. Even though we expected to see a lower incidence, owing to the high number of deliveries by cesarean section, we still observed a higher rate of intraventricular hemorrhage in the preeclampsia group (16 cases in the study group vs. 7 in the control, *p* = 0.085). Neutropenia and thrombocytopenia were more frequent in preterm newborns exposed to preeclampsia. Conclusions: The study shows that early preeclampsia increases the risk of complications in preterm neonates. RDS was more frequent in the exposed group than in the control group. The severity of preeclampsia correlates with hematological changes.

## 1. Introduction

Preeclampsia is defined as hypertension in pregnancy after the gestational age of 20 weeks associated with proteinuria (>300 mg/day), multiple organ dysfunction (renal, hepatic, neurological, and hematolog3e1ric involvement), or uteroplacental dysfunction potentially causing intrauterine growth restriction (IUGR) [1].

According to ACOG (American College of Obstetricians and Gynecologists), gestational hypertension is defined as hypertension with a systolic blood pressure of ≥140 mm Hg or a diastolic blood pressure of ≥90 mm Hg or both without proteinuria that develops after 20 weeks of gestation with a return to normal blood pressure after delivery [2]. Severe hypertension is defined by a systolic blood pressure of >160 mm Hg or a diastolic blood pressure of ≥110 mm Hg or both [2].

Preeclampsia is defined as hypertension, developed after 20 weeks of gestation and proteinuria. If proteinuria is absent, the new onset of any of the following has to be associated:

- Thrombocytopenia (platelets less than 100 × 10^9^/L);
- Renal failure (a serum creatinine level greater than 1.1 mg/dL, or doubling the baseline value);
- Liver function (liver transaminases that are more than 2 times the upper limit of normal value);
- Pulmonary edema;
- Central nervous disturbance (new-onset, severe headache, unresponsive to medication without an alternative diagnosis or visual symptoms-scotomata) [2].

Depending on the time of occurrence of the disease, there is an early form (onset before 34 weeks) and a late form (onset after 34 weeks of gestation). The early form of the disease is rarer, but with a higher incidence of neonatal complications and perinatal death. In the early form of the disease, abnormal placentation occurs, with the abnormal development of spiral arteries that remain with a narrow lumen, causing placental perfusion disorders as well as the release of inflammatory cytokines and proangiogenic factors that will trigger an endothelial response. This will generate the clinical picture and IUGR [3]. In the late form of the disease, vascular abnormalities are limited and much reduced compared to the early form. As a result, neonatal complications in this type of preeclampsia are rare or non-existent [1,4,5,6].

Preeclampsia increases the risk of renal failure, hepatic involvement, pulmonary edema, cerebral hemorrhage, and evolution towards eclampsia, with a risk of placental abruption and fetal death. In the newborn, placental hypoperfusion may cause IUGR and oligoamnios. The weight of neonates born to mothers with eclampsia is 5% lower compared to those born to mothers with normal blood pressure. In the early forms of the disease, birth weight is 23% lower compared to the value corresponding to the gestational age [1,6,7]. The mortality rate for newborns of mothers with preeclampsia is 5.2 per 1000, compared to 3.6 per 1000 for the population unaffected by preeclampsia [8,9].

This case-control study aims to evaluate the immediate effect of maternal preeclampsia on the morbidity and immediate clinical outcome of preterm newborns.

## 2. Materials and Methods

### 2.1. Study Design

We conducted a longitudinal case-control study in the neonatology department of the Clinic of Obstetrics and Gynecology I of the County Clinical Emergency Hospital, Cluj-Napoca. The study included preterm newborns with a gestational age of between 30 weeks and 34 weeks + 6 days, admitted between November 2017 and October 2019. The study was approved by the Institutional Review Board of the Iuliu Hatieganu University of Medicine and Pharmacy, Cluj Napoca, Romania.

### 2.2. Participants

The criteria for defining maternal preeclampsia were: hypertension after the gestational age of 20 weeks, associated with proteinuria. Proteinuria represents a loss of 300 mg in a 24-h urine collection or a protein/creatinine ratio of >0.3 in a single voided urine [2,10]. Based on the severity of the disease, preeclampsia is classified into “preeclampsia without severe features”, defined as hypertension (systolic blood pressure ≥140 mm Hg or diastolic blood pressure ≥90 mm Hg) with proteinuria and “preeclampsia with severe features”, defined as systolic blood pressure over 160 mmHg and diastolic blood pressure higher than 110 mmHg or both associated with end-organ dysfunction [9,11,12].

The threshold of severe hypertension in pregnancy is lower than in non-pregnant adults. Pregnant women are at higher risk of developing hypertensive encephalopathy at lower blood pressures than the normal adult population [7,12,13].

The study did not include preterm newborns with known chromosomal abnormalities or malformations diagnosed before or immediately after birth. The control group included preterm neonates matched for gestational age with the case group, whose mothers had normal blood pressure during pregnancy. In the current study, newborn complications and outcomes up to discharge from the NICU were followed up. The long-term outcome, especially the neurological development of the study group, is not part of this study.

In the study period, November 2017 and October 2019, out of 4935 admissions, we had 255 newborns with a gestational age of between 30 weeks and 34 weeks + days. Of them, 45 were identified as having maternal preeclampsia. For our study, 42 preterm neonates were enrolled who had complete data in their records. In the same age category, we enrolled 44 control patients who were not affected by preeclampsia.

### 2.3. Variables

In all the newborns included in the study, the incidence and severity of respiratory distress syndrome (RDS) were monitored. The RDS was defined as: clinical signs of retractions, grunting, flaring, and tachypnea associated with radiological findings, namely reticulogranular patterns, air bronchograms, and ground glass appearance.

The administration of surfactant was recorded in patients of both groups. The presence of IUGR, hypoglycemia, and intraventricular hemorrhage was analyzed. Also, we monitored the incidence of thrombocytopenia (normal range: 150,000–300,000/mm^3^) and leukopenia (normal range: 3000–30,000/mm^3^) in neonates exposed to maternal hypertension compared to unexposed neonates. The severity of RDS was classified based on the Silverman score into three forms: mild, moderate, and severe, depending on the clinical manifestations. In addition, the need for surfactants and the duration of respiratory support were also assessed.

Those newborns with weight, length, and cranial circumference below the 10th percentile on the growth curves for their gestational age were considered small for gestational age (SGA). The diagnosis of IUGR was established based on the clinical examination. It applies to neonates with clinical features of malnutrition and in-utero growth retardation, irrespective of their birth weight percentile. Large heads compared to the rest of the body, large anterior fontanelle, scaphoid abdomen, thin umbilical cord, decreased subcutaneous fat tissue and skeletal muscle mass, loose, dry skin, long fingernails, large hands and feet compared to body size, and hyper-alert infants are all typical clinical features of newborns with IUGR [14,15]. There are small differences, and there is frequently overlap between the two terms “IUGR” and “SGA”. The fetal growth restriction was considered as a single criterion, at a weight <3rd percentile.

The severity of cerebral hemorrhage was quantified by head ultrasound performed in the first 72 h of life. The ultrasound was carried out with a GE device, in two sections: sagittal and coronal, using the 8 Hz probe, by a neonatologist experienced in head ultrasound.

All neonates included in the study had pH gas value parameters evaluated within the first hour of life. We considered a pH value < 7 clinically significant in the first hour of life. Thrombocytopenia was defined as a platelet value lower than 150,000/mm^3^, and leukopenia was considered a value below 3000/mm^3^.

The data were obtained by analyzing the medical documents of the patients included in the study. It is an observational study using anonymized data from medical records. Women were informed at admission that their records could be used for the evaluation of medical practices and were provided with the option to opt-out of these studies.

### 2.4. Data Analysis

Categorical variables were presented by counts and percentages, while comparisons between two independent groups were made with the chi-square test or Fisher’s exact test (when the expected frequencies were low). Continuous skewed variables were presented by medians and interquartile ranges, while comparisons between two independent groups were performed using the Mann–Whitney U test. For all statistical tests, a significance level of 0.05 was used, and the two-tailed *p*-values were computed. All statistical analyses were carried out with the R environment for statistical computing and graphics (R Foundation for Statistical Computing, Vienna, Austria), version 4.0.2.

## 3. Results

### 3.1. Demographic Data and Perinatal Outcome

The demographic data analysis showed a higher mean birth weight in the unexposed group compared to the newborns exposed to maternal preeclampsia (Table 1).

Infants exposed to preeclampsia had lower birth weights than infants not exposed. There was a significant difference in SGA between exposed and unexposed infants, with all SGA infants in the exposed group (*p* < 0.001). There was no significant group difference in the incidence of asphyxia at birth (*p* = 0.673).

### 3.2. Delivery Mode

Most of the deliveries in the group of newborns exposed to preeclampsia were performed by cesarean section (Table 1).

### 3.3. Pathologies of the Study Group

We analyzed the incidence of neonatal complications in the studied groups (Table 2).

The frequency and severity of RDS were analyzed in both groups. It should be mentioned that the severe forms of RDS were two times more frequent in the group of newborns exposed to preeclampsia compared to those in the control group. Surfactant therapy was necessary in 16 cases (38.1%) of the group exposed to preeclampsia and only in five (11.36%) cases in the unexposed group. The severity of RDS required invasive respiratory support (synchronized intermittent mandatory ventilation-SIMV) and continuous positive airway pressure (CPAP) support, the duration of which was longer in the study group compared to the control group. Infants exposed to preeclampsia required invasive respiratory support –SIMV more often than the non-exposed ones. 

The incidence of intraventricular hemorrhage was significantly higher in the exposed infants to preeclampsia than in the non-exposed ones (*p* = 0.085). Hypoglycemia was present more often in infants exposed to preeclampsia; the difference between the groups was not statistically significant (*p* = 0.121). We did not find a significant group difference in pH gas value parameters (*p* > 0.05).

The incidence of thrombocytopenia and neutropenia was higher in the group exposed to preeclampsia (6 vs. 2 and 14 vs. 7, respectively), but the difference was not statistically significant. There was a relatively small number of thrombocytopenia cases in both groups, while neutropenia was more frequent than thrombocytopenia in both groups.

In the study group, the severe form of maternal preeclampsia was more frequent (30, 71.43%) than the mild form (12, 29.57%). Both thrombocytopenia and neutropenia occurred more often in cases of the severe form of maternal preeclampsia; the influence of preeclampsia severity on these values was not statistically significant (*p* = 0.655 and *p* = 0.719).

## 4. Discussion

We examined the association between maternal preeclampsia and preterm neonate outcomes compared to preterm newborns of the same gestational age unexposed to this maternal condition. Our cohort of 84 mother–preterm newborn pairs included 30 mothers with severe forms of preeclampsia and 12 cases with mild disease forms. The newborns in this study were exposed to the early form of preeclampsia, with an onset before 34 weeks.

Maternal preeclampsia and its complications (hepatic failure, renal failure, coagulation disorders) require the termination of pregnancy by cesarean section [11,14,16,17,18]. In early preeclampsia, the main factors that inform the decision to deliver by cesarean section are the maternal condition and the fetal status, which need to be considered against the benefits of prolonging the pregnancy for the newborn because a compromised intrauterine environment will exert a greater negative impact on the neonate than preterm birth [18,19,20]. In our case, 95% of the births in the study group were performed by cesarean section, a significantly higher proportion than in the control group (40 vs. 18). This is a common approach found in other studies. Rescue cesarean section is recommended when the maternal or fetal condition becomes stable [21,22,23,24,25].

The birth weight of the newborns was significantly lower in the case of exposure to preeclampsia (*p* < 0.001). In their study, Odegard et al. reported that the newborns of mothers with early preeclampsia had a 23% lower average birth weight compared to the weight considered adequate for gestational age [1]. In our study, there was a 32% difference in the birth weight between the neonates exposed and unexposed to preeclampsia. Fetal growth restriction increases the risk of poor neonatal outcomes. Certainly, fetal growth and development are influenced by several other factors other than maternal hypertension [26]. In a future study, it will be useful to analyze other risk factors impacting fetal growth that are associated with preeclampsia, such as maternal diet, socioeconomic status, or living conditions.

After birth, the association and severity of some diseases were monitored, as well as the frequency of some therapies in the group of neonates exposed to hypertension compared to those unexposed. Asphyxia at birth had a similar incidence in the two groups. Maternal preeclampsia did not influence the immediate transition of the newborns. The birth occurred in the preeclampsia group before severe fetal impairment developed, with an impact on adaptation to birth and on the transition to extrauterine life. Thus, it can be extrapolated that termination of pregnancy by cesarean section was adequate for the newborns of the case group. Tian et al. reported a low Apgar score more often in the case of newborns from mothers with pregnancy hypertension and preeclampsia, and the risk had an increasing trend with the progress of maternal hypertension than in the non-exposed population [27]. Maternal hypertension and preeclampsia increase the risk of a low Apgar score [27,28].

RDS was the most frequent disorder found in both groups. Preterm birth in association with preeclampsia more frequently causes more severe forms of RDS, which require surfactant therapy and a longer period of respiratory support [27,29,30]. In our study, the severe forms of the disease were two times more frequent in neonates of mothers with preeclampsia compared to unexposed newborns. Although preeclampsia is an important intrauterine stress factor that accelerates lung maturation, in the studied group it did not have such an effect. While the gestational age of the preterm neonates included in this study was over 30 weeks, 38.1% of these required surfactant administration, compared to 11% of those unexposed to preeclampsia. Moreover, the duration of respiratory support with SIMV and the support via CPAP ventilation were longer in the group of preterm neonates exposed to preeclampsia. The factors that might explain the higher severity of RDS are fetal suffering caused by maternal preeclampsia and possibly the antiangiogenic status that disturbs the lung development of preterm newborns of preeclamptic mothers [3,31,32,33]. This intrauterine environment changes because placental hypoperfusion induced by vascular remodeling in preeclampsia can also generate chronic pulmonary complications such as bronchopulmonary dysplasia with pulmonary hypertension [27,29,30,33,34]. Other studies describe the same higher incidence and also a higher need for respiratory support in the preterm population exposed to maternal hypertension and preeclampsia [27,33,35]. Long-term chronic hypoxia leads to respiratory disorders in neonates exposed during pregnancy to preeclampsia [25,26,27,28].

The incidence of mild forms of intraventricular hemorrhage (IVH) was higher in the study group, but this form of IVH resumed completely by the time the neonates reached 37 weeks of corrected age. Despite this, there were no statistically significant differences between the study and the control groups, even though we expected to see a lower incidence of IVH in the study group, owing to the high number of deliveries by cesarean section. Preeclampsia will cause immediate and long-term neurological problems. The study of Venkatesh et al. in a large cohort describes a higher incidence of IVH in preterm neonates below 34 weeks of gestation exposed to severe features of preeclampsia [33]. The long-term neurological outcome is marked by the higher frequency of cerebral palsy, but also more frequent autism. These are studies that found that cerebral palsy occurs more often if severe features of preeclampsia occur, but did not find a higher rate of autism or eating disorders [26,28].

Hypoglycemia associated with growth restriction and prematurity was present in the studied group in 38.1% of cases compared to 22.73% of the control group cases. All cases in the study had transient hypoglycemia that responded favorably to parenteral feeding with a glucose intake adequate for weight and gestational age. We explained the higher frequency of hypoglycemia in the group of newborns from preeclampsia mothers’ maternal pathology, which is an important stress factor for the fetus and the newborn, with an impact on their postnatal adaptation. In the reviewed literature, we found no data demonstrating an explanation by a different pathophysiological mechanism for the frequency and duration of hypoglycemia in this category of preterm neonates [33,36]. Immediate postnatal adaptation was similar in all newborns in the case group to that of preterm neonates in the control group.

Preeclampsia is known to be a determining factor in thrombocytopenia and neutropenia in newborns. In our study, we could not demonstrate a significant relationship between the incidence of thrombocytopenia and neutropenia and preeclampsia, although the number of cases presenting these changes was higher in preterm neonates with preeclampsia. Both neutropenia and thrombocytopenia were more frequent in preterm newborns whose mothers had severe forms of preeclampsia and had antepartum thrombocytopenia. In none of the cases did thrombocytopenia cause hemorrhagic complications or require platelet administration. Neutropenia represents a factor favoring infections. In the preterm newborns of the study group, there was no evidence of infectious risk induced by neutropenia. None of the neonates with neutropenia had neonatal sepsis. Indeed, the small sample size can be a reason for the lack of a statistically significant association. We had not performed sample size estimation before undertaking the study.

Finally, we analyzed the impact of fetal distress based on pH gas value parameters, evaluated within the first hour of life in both the study and the control groups. Metabolic acidosis was only detected in five (11.9%) newborns of the study group, with only two (4.76%) neonates in this group showing severe acidosis with a pH lower than 7.

The strength of our study lies in the analysis of complications associated with preeclampsia compared to the group unexposed to this risk factor. The comparison with a group of unexposed patients of the same gestational age allows for some pertinent conclusions to be drawn as well as a better understanding of how to approach preeclampsia cases. The fact that this study was conducted in one center allowed us to undertake a more rigorous analysis of the procedures used, which were closely monitored in both groups, being applied based on the criteria of the same protocol and being homogeneous except for the space criterion. However, data collected at a single site may limit its generalizability.

## 5. Conclusions

Early preeclampsia increases the risk of complications in preterm newborns. The high number of deliveries by cesarean section did not protect the newborns against intraventricular hemorrhage. The RDS was more frequent and severe in the preeclampsia group, even though we expected to see advanced pulmonary maturation due to exposure to an important intrauterine stress factor that accelerates lung maturation.

## Figures and Tables

**Table 1:** Demographic data and perinatal outcome.

Characteristics	Preeclampsia (*n* = 42)	Control (*n* = 44)	*p*-Value
Gestational age (weeks), median [IQR] (range from 28–34)	32 (30–34)	32 (30–34)	0.997
Sex (M), *n* (%)	17 (40.48)	28 (63.64)	0.032
Birth weight (g), median [IQR]	1274.5 (990–1852.5)	1895 (1490–2700)	<0.001
Delivery mode (C-section), *n* (%)	40 (95.24)	18 (40.91)	<0.001
Asphyxia, *n* (%)	3 (7.14)	2 (4.55)	0.673
SGA, *n* (%)	17 (40.48)	0 (0)	<0.001
IUGR, *n* (%)	15 (35.71)	5 (11.36)	0.008

M = male, SGA = small for gestational age, IUGR = intrauterine growth restriction, IQR = interquartile range.

**Table 2:** Pathologies of the study groups.

Characteristics	Preeclampsia (*n* = 42)	Control (*n* = 44)	*p*-Value
Respiratory distress syndrome, *n* (%)			0.482
No	10 (23.81)	12 (27.27)	
Mild	11 (26.19)	12 (27.27)	
Moderate	11 (26.19)	15 (34.09)	
Severe	10 (23.81)	5 (11.36)	
Surfactant, *n* (%)	16 (38.1)	5 (11.36)	0.004
CPAP, *n* (%)			0.041
No	14 (33.33)	13 (29.55)	
24	6 (14.29)	9 (20.45)	
36	1 (2.38)	0 (0)	
48	10 (23.81)	2 (4.55)	
72	2 (4.76)	9 (20.45)	
>72	9 (21.43)	11 (25)	
SIMV, *n* (%)			0.009
No	27 (64.29)	39 (88.64)	
24	3 (7.14)	1 (2.27)	
36	0 (0)	1 (2.27)	
48	6 (14.29)	0 (0)	
>72	6 (14.29)	3 (6.82)	
IVH, nr (%)			0.085
Gr. I	16 (38.1)	7 (15.91)	
Gr. II	2 (4.76)	2 (4.55)	
Gr. III	1 (2.38)	1 (2.27)	
No	23 (54.76)	34 (77.27)	
Hypoglycemia (< 40 mg/dL), *n* (%)	16 (38.1)	10 (22.73)	0.121
pH < 7, *n* (%)	2 (4.76)	1 (2.27)	0.612
HCO_3_ < 15 mEq/L, *n* (%)	5 (11.9)	1 (2.27)	0.106
BE < −4 mmol/L, *n* (%)	19 (45.24)	23 (52.27)	0.514
Thrombocytopenia, *n* (%)	6 (14.29)	2 (4.55)	0.152
Neutropenia, *n* (%)	14 (33.33)	7 (15.91)	0.06

CPAP = continuous positive airway pressure, SIMV = synchronized intermittent mandatory ventilation, IVH = intraventricular hemorrhage, HCO_3_ = bicarbonate, BE = basal excess, *n* = number.

## Data Availability

All data generated or analyzed during this study are included in this published article.
